# Sleep duration, health status, and subjective well-being: a population-based study

**DOI:** 10.11606/S1518-8787.2018052000602

**Published:** 2018-09-03

**Authors:** Margareth Guimarães Lima, Marilisa Berti de Azevedo Barros, Maria Filomena Ceolim, Edilson Zancanella, Tânia Aparecida Marchiori de Oliveira Cardoso

**Affiliations:** I Universidade Estadual de Campinas. Faculdade de Ciências Médicas. Departamento de Saúde Coletiva. Campinas, SP, Brasil; II Universidade Estadual de Campinas. Faculdade de Enfermagem. Campinas, SP, Brasil; III Universidade Estadual de Campinas. Faculdade de Ciências Médicas. Departamento de Oftalmologia/Otorrinolaringologia. Campinas, SP, Brasil; IV Universidade Estadual de Campinas. Faculdade de Ciências Médicas. Departamento de Neurologia. Campinas, SP, Brasil

**Keywords:** Sleep, Comorbidity, Chronic Disease, Sickness Impact Profile, Socioeconomic Factors, Life Style, Quality of Life

## Abstract

**OBJECTIVE:**

To evaluate, in a population-based approach, the association of extreme sleep duration with sociodemographic factors, health, and well-being.

**METHODS:**

We analyzed the data from the 2014/2015 Health Survey in the city of Campinas, State of São Paulo, Brazil (ISACamp), performed with 1,969 individuals (≥ 20 years old). Associations between the independent variable and short (≤ 6 hours) and long (≥ 9 hours) sleep were determined using the Rao-Scott chi-square test. The analyses were adjusted with multinomial logistic regression models.

**RESULTS:**

Men, individuals aged 40 to 59, those with higher schooling, those who have one (OR = 1.47, 95%CI 1.02–2.12), two (OR = 1.73, 95%CI 1.07–2.80), or three or more (OR = 1.62, 95%CI 1.16–2.28) chronic diseases, and those with three or more health problems (OR = 1.96, 95%CI 1.22–3.17) were more likely to have a short sleep. The chance of long sleep was higher in widowers and lower in those who have more years of schooling, with higher income, worked, lived with more residents at home, and reported three or more diseases (OR = 0.68, 95%CI 0.48–0.97) and health problems. The chance of either short (OR = 2.41, 95%CI 1.51–3.87) or long sleep (OR = 2.07, 95%CI 1.23–3.48) was higher in unhappy individuals.

**CONCLUSIONS:**

These findings highlight the higher chance of short sleep duration among men, among persons in productive age, and among those with a higher level of schooling in a Brazilian city. The association of short sleep with comorbidities and the association of happiness with extremes of sleep duration were also important results to understand the relation of sleep duration with health and well-being.

## INTRODUCTION

Questions about sleep duration have been incorporated into health surveys in the United States^1^, England^2^, and Japan^3^ since the early 1980s. Studies have shown that short and long sleep are associated with all-cause mortality risk^2^ ^,^ ^3^ and the prevalence of diseases, such as diabetes, hypertension, depression, rheumatic diseases, osteoporosis^1^ ^,^ ^4^ ^,^ ^5^, and disabilities^6^. Considering the importance of sleep-related health issues, sleep duration has been incorporated into the indicators and goals to improve the American population health by 2020^7^.

Socioeconomic and demographic conditions play an important role on sleep duration. Studies point out that age, marital status, work, income, and schooling are associated with short and long sleep pattern^4^ ^,^ ^8^ ^,^ ^9^, and cultural conditions, such as region of residence, may influence this relationship^9^ ^,^ ^10^.

Multiple chronic conditions are an important subject in public health^11^ and they may also be associated with sleep outside the median variation. According to a population-based study, performed in a Brazilian city, the report of three or more chronic diseases increased twice the chance of short sleep, in relation to individuals without morbidities^4^.

In addition, sleep restriction is related to changes in mood and well-being^12^. Studies have shown associations of states of affect, such as happiness, positive feelings, and vitality, with sleep quality^13^ and sleep duration^14^. There seems to be a bidirectional association between well-being and sleep: on the one hand, sleep disturbances negatively affect well-being and positive affects and, on the other hand, the better state of well-being promotes a better sleep quality^13^ ^,^ ^15^ ^,^ ^16^. Happiness and life satisfaction are important indicators of subjective well-being^17^, with particularities in their concepts: life satisfaction is more objective and encompasses an evaluation process; happiness is a more subjective process and is related to positive experiences^18^.

The reasons why short sleep is associated with worse health conditions and diseases are evident, especially considering how sleep time acts on the daily restoration and metabolic, endocrine, immune, and inflammatory functions^19^ ^,^ ^20^. However, the mechanisms that explain the associations of long sleep with morbidity, mortality, and well-being are still unclear or unknown^19^.

Therefore, we have hypothesized that sleep duration patterns vary according to demographic and socioeconomic factors and illness, including comorbidity and emotional problems, and they may have an important relation with subjective well-being.

To date, especially in Brazil and Latin America, there are few research studies focusing on the prevalence of short and long sleep and the relation with comorbidities and well-being in a population-based approach. Indeed, globally, studies about the association between sleep duration and happiness are scarce, especially those assessing long sleep. In this sense, our study aims to analyze associations of sleep duration with demographic and socioeconomic conditions, multiple chronic diseases, multiple health problems, and subjective well-being in the adult population of Campinas, State of São Paulo, Brazil.

## METHODS

This is a cross-sectional, population-based study carried out with data from the Health Survey of the Municipality of Campinas, State of São Paulo, Brazil (ISACamp 2014/2015). The survey was performed with the non-institutionalized population living in the urban areas of the city, in 2014 and 2015, using probabilistic sample in two stages. In the first stage, 70 census tracts were drawn, proportionally to the number of residences (14 census tracts in each of the five health districts of Campinas). In the second stage, households were drawn. A sample of 1,000 individuals was defined for the age group of 10 to 19 years old and 60 years old or more. For the adults (aged 20 to 59 years), the defined sample was 1,400 individuals. With this size, a prevalence of 0.50 (maximum variability for the frequency of events studied) could be estimate, with a 95% confidence level, sampling error of five percentage points, and considering a design effect equal to two.

Based on the 2010 Census, a probability of the number of persons, who would live at each home by age domain, was estimated for five districts. The initial sample size of 1,000 individuals for adolescent and elderly and 1,400 adults was divided by person/household ratio, which determined the number of residences to be visited as 2,898 for adolescents, 950 for adults, and 3,326 for older adults already considering the non-response rates of 27%, 22%, and 20%, respectively. All residents from each household of the selected age group were interviewed. This study only analyzed data from persons aged 20 years or over.

The ISACamp 2014 questionnaire was divided into 11 thematic blocks, and most of the questions were closed and pre-coded. The interviews were conducted face to face by trained and supervised interviewers using a tablet computer.

The dependent variable of the study was sleep duration, calculated from four questions: (1) “What time do you usually go to bed?” (2) “When you go to sleep, do you take a long time to fall asleep?” (3) “How long do you take to fall asleep, approximately?” (4) “What time do you usually wake up in the morning?”. Information on these questions was obtained for weekdays and weekend days. Thus, we constructed the indicator of sleep duration weighted for all days of the week. We discounted the reported time to fall asleep, relative to sleep onset latency. The variable was categorized into six hours or less (short sleep), seven to eight hours (median sleep), and nine hours or more (long sleep).

The independent variables of the study were the following ones:

- Demographic and socioeconomic conditions, encompassing sex (female; male), age group (20–39; 40–59; 60 or more), marital status (married or living together; separated/divorced; widowed; never married), and race (white; black or brown [other races were excluded because of the small number]). We assessed number of children (0–4; 5–8; 12 or more), work (yes; no), income (< 1; 1–2; 3 or more minimum wages), schooling (0–4; 5–8; 9–11; 12 or more years of schooling), and number of residents in the household (1; 2; 3 or more). The presence or not of health insurance was also assessed.
- Variables of illness: number of chronic diseases, based on the question whether a physician or health care professional had already diagnosed hypertension, diabetes, angina, infarction, arrhythmia, cancer, arthritis, rheumatism or arthrosis, asthma or asthmatic bronchitis, chronic emphysema or bronchitis, rhinitis, sinusitis, repetitive strain injuries or work-related musculoskeletal disease (RSI/WRMD), stroke, high cholesterol, or spinal disease. We added up all the diseases reported by the interviewee, categorizing them into none, one, two, and three or more. We also assessed reported chronic health problems, corresponding to complaints or symptoms about migraine or headache, back pain, allergy, emotional problems, vertigo, and urinary problems. This variable was categorized into none, one or two, and three or more. Common mental disorders (CMD) were assessed by the Self-Reporting Questionnaire (SRQ-20), and we considered CMD present with the score of eight points or more^21^.
- Subjective well-being variables were life satisfaction (categorized as very satisfied or slightly satisfied, and not satisfied) and feeling of happiness in the last four weeks, categorized into always or most of the time, some of the time, hardly ever, or never happy.

We estimated the prevalence and 95% confidence intervals (95%CI) of short, long, and normal sleep according to the independent variables and we tested the differences using the Rao-Scott chi-square test (chi-square test, suitable for the complex sample of the study). Furthermore, we estimated the odds ratio with 95%CI using multinomial logistic regression models. We developed a hierarchical model for both, short and long sleep, performed by backward stepwise regression in three stages. In the first stage, demographic and socioeconomic variables with p < 0.20, in the bivariate analysis, were taken to the regression and those with p < 0.05 remained in the model. In the second stage, we added, on the model, the variables of disease and illness to the demographic and socioeconomic variables that remained associated in the first stage. Finally, in a third stage, we added the variables of well-being to the variables remaining in the first and second model. This hierarchical model was performed considering social factors as distal variables, which can lead to illness and poor well-being, and the variables located in the three stages are associated with sleep duration outside the normal pattern.

We performed the analyses with the statistical software Stata 14.0, which considers the necessary weights for the complex sampling design. The research ethics committee, Process 409.714 of September 30, 2013, CAAE 20547513.2.0000.5404, approved the ISACamp 2014/2015 project.

## RESULTS

A total of 1,969 individuals were studied. Mean age was 43.7 years (95%CI 42.3–45.2), and 52.7% of the total (95%CI 49.7–55.8) were female. There was a loss of 8.7% because of refusals and other losses among the households selected for the interviews. Among the adults and older adults identified to be interviewed, 21% refused to be part of the research and 1.8% was lost for other reasons.

The mean sleep duration of the population was 7.67 hours (95%CI 7.56–7.78): 7.60 for adults (20–59 years) and 8.00 for older adults. The percentages of sleep duration were 4.1% for sleep < 4 hours, 6.4% for five hours, 17.2% for six hours, 27.8% for seven hours, 25.1% for eight hours, 12.1% for nine hours, and 7.3% for 10 hours or more.

The demographic and socioeconomic factors associated with sleep duration were sex (with the highest prevalence of short and long sleep in men and women, respectively), and age group (with the highest frequency of long sleep among persons aged 60 or over). Separated or divorced individuals presented the highest prevalence of short sleep, and widowers, of long sleep. Schooling was also associated with sleep: the higher prevalence of long sleep can be observed in the lower level and the higher percentage of short sleep is in the highest level of schooling. According to income, the highest prevalence of long sleep was among individuals with lower income. Living alone or with one or more person and no work were associated with long sleep. Short sleep was observed in those who had health insurance (Table 1).

**Table 1:** Sleep duration according to demographic and socioeconomic variables in the adult population (20 years old or more). Campinas, State of São Paulo, Brazil, 2014–2015.

Variable		Sleep Duration (%)			p*
		≤ 6 hours (n = 478)	7 to 8 hours (n = 1020)	≥ 9 hours (n = 501)	
Sex					< 0.01
	Female	23.9	53.7	22.4	
	Male	31.9	51.9	16.2	
Age group (years)					< 0.01
	20 to 39	26.3	53.4	20.3	
	40 to 59	33.6	53.5	13.0	
	60 or more	20.1	50.3	29.7	
Marital status					0.01
	Married/Living together	27.6	54.0	18.4	
	Separated/Divorced	36.2	47.3	16.5	
	Widower	20.5	44.7	34.8	
	Never married	27.1	54.6	18.4	
Race					0.40
	White	28.5	52.7	18.8	
	Black and brown	25.5	53.1	21.4	
Schooling (years)					< 0.01
	0 to 3	18.8	52.0	29.2	
	4 to 8	23.8	51.9	24.3	
	9 to 11	28.5	52.5	19.0	
	12 or more	34.1	54.5	11.4	
*Per capita* family income					< 0.01
	< 1 minimum wage	24.3	51.6	24.1	
	1 to 2	31.6	50.3	18.2	
	3 or more	23.2	63.6	13.2	
Work status					< 0.01
	Not working	22.0	47.4	30.7	
	Working	31.0	55.8	13.2	
Number of inhabitants in the residence					0.02
	1	28.7	44.6	26.7	
	2	23.1	51.3	25.6	
	3 or more	28.7	54.0	17.3	
Number of children					0.12
	0	25.3	55.7	19.0	
	1	30.3	52.1	17.6	
	2 or more	26.0	50.6	23.4	
Health insurance					< 0.01
	No	24.1	52.2	23.7	
	Yes	31.7	53.5	14.8	

* Rao-Scott chi-square test.

A higher percentage of short sleep was observed in those reporting one or more diseases or health problems. Happiness was also associated with sleep duration: we observed the highest prevalence of long and short sleep in persons who feel unhappy (Table 2).

**Table 2:** Sleep duration according to health and well-being in the adult population (20 years old or more). Campinas, State of São Paulo, Brazil, 2014–2015.

Variable		Sleep duration (%)			p*
		≤ 6 hours (n = 478)	7 to 8 hours (n = 1020)	≥ 9 hours (n = 501)	
Number of chronic diseases					0.01
	None	20.1	56.6	23.4	
	One	29.8	53.9	16.3	
	Two	32.7	49.2	18.1	
	Three or more	30.6	50.2	19.2	
Number of health problems					0.02
	None	20.5	58.3	21.2	
	One or two	28.8	52.5	18.6	
	Three or more	33.1	47.6	19.3	
Common Mental Disorders (SRQ-20)					0.12
	No	27.0	53.9	19.1	
	Yes	32.7	45.0	22.3	
Satisfaction with life					0.13
	Very satisfied	28.0	54.2	17.8	
	More or less or not at all satisfied	27.4	50.2	22.5	
Time of happiness (last 4 weeks)					< 0.01
	Always/Most of the time	27.2	54.5	18.3	
	Some of the time	25.3	54.3	20.4	
	Hardly ever or never	37.9	34.7	27.4	

* Rao-Scott chi-square test.

In the multivariate analysis, in the first stage of the regression models, the demographic and socioeconomic variables that remained associated with short sleep were: sex (higher chance among men), age (higher chance among those aged 40 to 59 years), and schooling [higher chance in higher education (OR = 1.73)]. In the second stage we observed the highest chance of short sleep among individuals who reported one (OR = 1.47), two (OR = 1.73), three or more chronic diseases (OR = 1.62), and one or two (OR = 1.36) and three or more (OR = 1.96) chronic health problems. In the third stage, we verified an association of short sleep duration with happiness (OR = 2.41) (Table 3).

**Table 3:** Odds ratio (OR) and 95%CI of short sleep duration according to sociodemographic variables, health problems, and well-being. Campinas, State of São Paulo, Brazil, 2014–2015.

Variable		≤ 6 hours					
		Stage 1^a^		Stage 2^b^		Stage 3^c^	
		OR	95%CI	OR	95%CI	OR	95%CI
Sex							
	Female	1		1		1	
	Male	1.39	1.05–1.85	1.57	1.16–2.13	1.54	1.13–2.09
Age (years)							
	20 to 39	1		1		1	
	40 to 59	1.43	1.04–1.96	1.28	0.93–1.78	1.31	0.95–1.82
	60 or more	1.00	0.75–1.33	0.85	0.62–1.18	0.86	0.62–1.19
Schooling (years)							
	0 to 3	1		1		1	
	4 to 8	1.15	0.74–1.81	1.07	0.69–1.65	1.11	0.73–1.71
	9 to 11	1.42	0.84–2.42	1.36	0.80–2.33	1.45	0.87–2.44
	12 or more	1.73	1.08–2.75	1.60	0.98–2.62	1.77	1.10–2.84
Number of chronic diseases							
	None			1		1	
	One			1.47	1.02–2.12	1.47	1.00–2.17
	Two			1.73	1.07–2.80	1.75	1.08–2.84
	Three or more			1.62	1.16–2.28	1.59	1.11–2.29
Number of health problems							
	None			1		1	
	One or two			1.36	1.00–1.84	1.52	1.05–2.22
	Three or more			1.96	1.22–3.17	1.75	1.03–2.96
Time of happiness (last 4 weeks)							
	Always/Most of the time					1	
	Some of the time					0.81	0.53–1.23
	Hardly ever or never					2.41	1.51–3.87

a Multinomial regression model analyzing all socioeconomic and demographic variables with p < 0.20 in bivariate analysis.

b Multinomial regression model adjusted for sociodemographic variables.

c Multinomial regression model adjusted for all variables of the table.

Association with long sleep was observed in age, with a lower chance in individuals over 40 years of age. In persons aged 60 years or more, the association was reversed (in relation to the bivariate analysis) because of the variables of schooling, work, and number of residents. Other variables associated with long sleep in the first model were marital status [higher chance in widowers (OR = 1.50)], schooling, income, work, and number of residents at home; we verified lower OR for long sleep in the highest level of schooling and income, in those who work, and among individuals living with three or more residents. The chance of long sleep was lower in individuals with three or more chronic diseases in the second stage (OR = 0.68). Finally, we observed an association of unhappiness and long sleep in the third stage (OR = 2.07) (Table 4).

**Table 4:** Odds ratio (OR) and 95%CI of long sleep duration according to demographic and socioeconomic conditions, chronic diseases, and well-being. Campinas, State of São Paulo, Brazil, 2014–2015.

Variable		≥ 9 hours					
		Stage 1^a^		Stage 2^b^		Stage 3^c^	
		OR	95%CI	OR	95%CI	OR	95%CI
Sex							
	Female	1		1		1	
	Male	0.87	0.62–1.21	0.80	0.55–1.16	0.81	0.56–1.19
Age							
	20 to 39	1		1		1	
	40 to 59	0.46	0.29–0.74	0.47	0.28–0.78	0.45	0.28–0.74
	60 or more	0.55	0.33–0.91	0.56	0.32–0.98	0.53	030–0.93
Marital status							
	Married/Living together	1		1		1	
	Separated/Divorced	1.00	0.58–1.79	0.91	0.53–1.54	0.90	0.53–1.51
	Widowed	1.50	1.03–2.20	1.55	1.07–2.24	1.54	1.05–2.25
	Never married	0.98	0.59–1.62	0.89	0.55–1.43	0.86	0.53–1.40
Schooling (years)							
	0 to 3	1		1		1	
	4 to 8	1.09	0.72–1.65	0.99	0.64–1.53	1.02	0.67–1.54
	9 to 11	0.87	0.56–1.36	0.85	0.54–1.36	0.90	0.57–1.40
	12 or more	0.54	0.30–0.98	0.49	0.27–0.90	0.52	0.29–0.95
Income							
	< 1 minimum wage	1		1		1	
	1 to 2	0.84	0.61–1.15	0.82	0.58–1.15	0.83	0.58–1.17
	3 or more	0.49	0.29–0.85	0.46	0.26–0.81	0.47	0.26–0.85
Work							
	Not working	1		1		1	
	Working	0.39	0.28–0.55	0.38	0.27–0.53	0.40	0.28–0.56
Number of inhabitants in the residence							
	1	1		1		1	
	2	0.98	0.57–1,68	0.97	0.56–1.66	0.99	0.58–1.69
	3 or more	0.48	0.28–0.80	0.43	0.26–0.70	0.43	0.26–0.69
Number of chronic diseases							
	None			1		1	
	One			0.72	0.46–1.09	0.72	0.47–1.10
	Two			0.86	0.48–1.55	0.88	0.48–1.59
	Three or more			0.68	0.48–0.97	0.66	0.45–0.96
Time of happiness (last 4 weeks)							
	Always/Most of the time					1	
	Some of the time					1.18	0.77–1.80
	Hardly ever or never					2.07	1.23–3.48

a Multinomial regression model analyzing all socioeconomic and demographic variables with p < 0.20 in bivariate analysis.

b Multinomial regression model adjusted for sociodemographic variables.

c Multinomial regression model adjusted for all variables of the table.

## DISCUSSION

According to this study, the mean sleep duration of the Campinas adult population was 7.7 hours, which corresponds to 7 hours and 42 minutes. The prevalence of mean sleep duration was 52.8%, which is lower than that found in the National Health Interview Survey (NHIS), in the United States, of 62.4% in 2009 in the population aged 18 years and over^22^, but it was similar to the percentage of 57.2% from the 2014 Behavioral Risk Factor Surveillance System (BRFSS)^8^.

Considering the impact of sleep deprivation on health^20^, we highlight that 34.992 persons in the city of Campinas sleep only 4 hours or less, and approximately 236,410 persons do not reach 7 hours of sleep. Our research detected the highest chance of short sleep in males, which is similar to the results of the NHANES^23^. However, other studies have found no difference by sex^8^ ^,^ ^9^. Individuals aged 40 to 59 years also had the highest chance of short sleep compared to younger ones. It is possible that these results are related to work as the population in this age group, mainly males, consists of formal workers, and there is a trend of short sleepers among workers, especially those with more working hours^24^.

In relation to long sleep, the chance decreased in individuals aged 40 and over. A previous study conducted in Campinas has also found the lowest chance of long sleep in older individuals^4^. This result may be due to decreased slow wave sleep and sleep efficiency with age^9^.

Socioeconomic status is also one of the factors associated with sleep duration. Similar to other studies^4^ ^,^ ^25^, the chance of long sleep was lower in the higher levels of schooling and income; however, in contrast to other findings^8^ ^,^ ^10^ ^,^ ^23^, our study found a higher chance of short sleep in the population with higher level of schooling (12 years or more). In the research of Lima et al.^4^, also carried out in Campinas, in 2008, no significant associations were found between short sleep and level of schooling. The association of short sleep and high socioeconomic level may be common in more developed areas^26^. It is possible that the type of work of the persons with higher level of schooling is related to administrative positions that require responsibilities and goals, which can be associated with physical and mental problems and affect the sleep duration^27^. However, a short sleep may also be an individual choice to take advantage of the time for work, leisure activities, or social relations.

According to our research, living with three or more individuals decreases the chance of long sleep. Less work overload and less noise may provide a better environment for sleep.

Considering the comorbidities, the study detected higher chance of short sleep among individuals with two or more chronic diseases and two or more complains or health problems. Authors have observed the relationship of chronic disease with short sleep under two directions: morbidity affecting sleep or, in the other hand, sleep deprivation leading to the occurrence of a disease^1^ ^,^ ^5^. Physical and emotional discomfort because of illness can introduce an important impact on sleep duration^9^. In addition, persons with three or more chronic diseases have the lowest chance of long sleep. Studies on morbidities and long sleep are less consistent and the biological explanation for these associations is not so clear^19^.

Happiness had a U-shaped association with sleep duration, with a higher chance of both long and short sleep in unhappy individuals. The association of positive affects, subjective well-being, and optimism with less sleep disturbances is reported in other studies. Ong et al.^13^, in a meta-analysis, although evaluating only few valid studies, have found consistency of association between positive affects and better sleep. In a cross-sectional analysis, authors have found a moderate correlation between sleep quality with mood and optimism^16^, as well as an inverse association between positive affects and hedonic well-being and sleep problems^15^. Steptoe et al.^15^ have found that the association between positive affect and sleep problems was independent of psychological distress. Subjective well-being or positive experiences improve health, may decrease the effects of stress^28^, and improve social relations and emotional support^15^. Therefore, these factors can play an important role on sleep duration.

Our research has some limitations. Measurements of sleep duration and chronic illness are self-reported, which may lead to information and memory bias; however, the mentioned measures of sleep duration have a good correlation with the objective ones^29^, and several studies have used them^1^ ^,^ ^2^ ^,^ ^4^ ^,^ ^9^. The information on happiness was obtained by single questions, but a unique question can be valid for use in large-scale^30^. It is also necessary to consider the characteristic of the cross-sectional study design, which does not indicate causal relationship. In this sense, regarding the reported happiness being associated with sleep duration, it is important to consider that sleep outside the normal patterns could lead to this feeling, or that it is necessary to pay special attention to unhappier individuals, in order to promote a healthy sleep.

On the other hand, our study has strengths. It was carried out with a representative sample of an urban population. The study used an indicator of multiple chronic diseases to verify the association of comorbidities with sleep duration, which are scarce in the literature on the subject. There are also few studies about sleep duration and happiness, and the analysis of these feelings has gained space in health research because of the importance of considering the health/illness process and well-being. Finally, there are few population-based studies in Brazil and Latin America on sleep duration, mainly analyzing extreme sleep duration.

Our findings revealed the higher chance of short sleep among men, among those in productive age, and in the highest level of schooling, which show how these subgroups of the population need better awareness to avoid sleep deprivation. Another important result was the association of short sleep with concomitance of chronic disease and health problems, which suggests that comorbidities should be controlled and the sick population should receive more care in relation to sleep duration. Furthermore, we observed an association of subjective well-being with sleep outside mean patterns, even after adjusting for several variables. Considering that research studies on this subject are scarce, other studies are needed to clear this relationship.
